# A Self-Renewing Biomimetic Skeletal Muscle Construct Engineered using Induced Myogenic Progenitor Cells

**DOI:** 10.1002/adfm.202300571

**Published:** 2023-09-27

**Authors:** Inseon Kim, Seunghun S. Lee, Adhideb Ghosh, Stephen J. Ferguson, Ori Bar-Nur

**Affiliations:** Laboratory of Regenerative and Movement Biology, Department of Health Sciences and Technology, ETH Zurich, Schwerzenbach 8603, Switzerland; Institute for Biomechanics, Department of Health Sciences and Technology, ETH Zurich, Zurich 8092, Switzerland; Laboratory of Regenerative and Movement Biology, Department of Health Sciences and Technology, ETH Zurich, Schwerzenbach 8603, Switzerland; Functional Genomics Center Zurich, ETH Zurich and University of Zurich, Zurich 8057, Switzerland; Institute for Biomechanics, Department of Health Sciences and Technology, ETH Zurich, Zurich 8092, Switzerland; Laboratory of Regenerative and Movement Biology, Department of Health Sciences and Technology, ETH Zurich, Schwerzenbach 8603, Switzerland

**Keywords:** induced myogenic progenitor cells, myogenic differentiation, skeletal muscle regeneration, tissue engineering

## Abstract

Skeletal muscle represents a highly organized tissue that primarily regenerates by myogenic stem cells. Mimicking an in vitro skeletal muscle differentiation program that contains self-renewing muscle stem cells and aligned myotubes is considered challenging. This study presents the engineering of a biomimetic muscle construct that can self-regenerate and produce aligned myotubes using induced myogenic progenitor cells (iMPCs), a heterogeneous culture consisting of skeletal muscle stem, progenitor, and differentiated cells. Utilizing electrospinning, polycaprolactone (PCL) substrates are fabricated to facilitate iMPC-differentiation into aligned myotubes by controlling PCL fiber orientation. Newly-conceived constructs contain organized multinucleated myotubes alongside self-renewing stem cells, whose differentiation capacity is augmented by Matrigel supplementation. Furthermore, this work utilizes single-cell RNA-sequencing (scRNA-seq) to demonstrate that iMPC-derived constructs faithfully recapitulate a step-wise myogenic differentiation program. Notably, when subjected to a damaging myonecrotic agent, self-renewing stem cells rapidly differentiate into aligned myotubes within the constructs, akin to skeletal muscle repair in vivo. Finally, this study demonstrates that the iMPC derivation protocol can be adapted to engineer human myoblast-derived muscle constructs containing aligned myotubes, showcasing potential for translational applicability. Taken together, this work reports a novel in vitro system that mirrors myogenic regeneration and skeletal muscle alignment for basic research and regenerative medicine.

## 1 Introduction

Body movements are governed by voluntary contractions of multinucleated muscle fibers (or myofibers), the functional component of skeletal muscle tissue.^[1]^ Due to its role in generating locomotion, skeletal muscle tissue displays a unique architecture comprised of highly organized myofibers, which enable muscles to contract and produce movement in a desired direction.^[1]^ In response to injury or disease, skeletal muscle tissue can rapidly regenerate by resident stem cells termed satellite cells (SCs).^[2,3]^ These cells reside between the basal lamina layer and the sarcolemma, and are typically found in a quiescent state primed for activation.^[2]^ Furthermore, SCs express high level of the transcription factor paired box 7 (Pax7) and exhibit elevated activity of the Notch signaling pathway.^[4,5]^ Upon insult, SCs play a pivotal role in restoring muscle homeostasis by forming new myofibers or repairing damaged ones.^[2,6]^ During this process, quiescent SCs (QSCs) are activated to form a proliferative cell population that can either self-renew and return to quiescence, or differentiate into committed progenitors and fusion-competent myocytes that merge with damaged myofibers for tissue repair.^[2,6]^

The isolation of SCs from muscles and their serial passaging in vitro as proliferative myoblasts has been central to the study of myogenesis.^[7,8]^ Typically, primary or immortalized myoblasts such as C2C12 are cultured in high serum containing medium and differentiate into myotubes by serum withdrawal, thus permitting the study of skeletal muscle formation in vitro.^[7,8]^ However, this assay typically gives rise to disorganized myotubes in the culture dish, rendering tissue engineering a highly desirable approach to produce aligned and functional skeletal muscle tissue-like constructs.^[9–11]^ Indeed, in past decades a multitude of studies reported on ordered architecture construction of skeletal muscle cells by using a variety of fabrication techniques including 3D printing,^[12]^ micropatterning,^[13]^ acoustic patterning^, [14,15]^ magnetic actuation,^[16]^ filamented lightbeam biofabrication,^[17]^ and electrospinning.^[10,11,18,19]^ These efforts were harnessed to produce organized skeletal muscle constructs, however most often reported only on production of aligned myotubes in the absence of self-renewing muscle stem cells, thus limiting the study of myogenesis in biomimetic constructs.^[11,19]^

Electrospinning is a high-throughput scaffold fabrication technique that enables production of nanofiber membrane apt for cell growth.^[20]^ This technique facilitates orientation control of electrospun nano-scaled fibers on the substrate, guiding cellular growth akin to 3D bioprinting.^[10]^ As a consequence of these attributes, electrospun substrates have been utilized to produce highly aligned myotubes using multiple materials including decellularized extracellular matrix (ECM),^[21,22]^ synthetic or natural polymers such as polyurethane,^[23]^ poly(*ɛ*-caprolactone) (PCL),^[27]^ poly(lactic*-co-*glycolic acid),^[19]^ fibrin/alginate,^[25]^ and gelatin.^[11,26]^ However, incorporation of self-renewing muscle stem cells into electrospun nano-scaled fibers that can regenerate muscle fibers has seldom been reported.^[10,11]^

To date, a variety of myogenic cells have been used for skeletal muscle tissue engineering purposes, predominantly immortalized myoblasts such as C2C12 or primary myoblasts.^[9–11]^ Yet, these myogenic cells do not faithfully mirror self-renewing SCs, and myoblasts tend to lose differentiation capacities with extended passaging.^[27]^ As an alternative, several studies have used pluripotent stem cell (PSC)-derived myogenic precursors, which share cellular features with muscle stem cells and can differentiate into myotubes.^[28–32]^ However, PSC-derived myogenic precursors and derivative myotubes may carry embryonic attributes, and oftentimes require intricate and lengthy differentiation protocols which may give rise to heterogeneous cultures containing multiple cell types.^[31,33,34]^

An alternative myogenic cell source suitable for skeletal muscle engineering entails induced myogenic progenitor cells (iMPCs), which are directly reprogrammed from fibroblasts by myogenic transcription factors, often in the presence of defined small molecules.^[35–41]^ Several recent studies reported on production of iMPCs by transient expression of the canonical myogenic transcription factor MyoD in conjunction with the small molecules Forskolin, RepSox, and CHIR99021 (F/R/C).^[35,42–44]^ Notably, iMPC cultures are composed of disorganized multinucleated and contractile myotubes, in addition to self-renewing muscle stem cells that can expand extensively while maintaining differentiation capacity.^[35]^ Furthermore, unlike conventional myoblasts, muscle stem cells present in iMPCs share several attributes with in vivo proliferating SCs, thus serving as a potential superior cell source for skeletal muscle engineering.^[42,43]^ Moreover, iMPCs require a relatively short time to produce and demonstrate cellular heterogeneity of regenerating skeletal muscle cells.^[42]^ However, whether iMPCs produced with MyoD and F/R/C supplementation can recapitulate a myogenic differentiation program in a 3D microenvironment and produce aligned myotubes, similar to muscle fibers in vivo, has not been investigated. Herein we set out to engineer an aligned skeletal muscle construct (SMC) by growing murine iMPCs on electrospun PCL substrates, and in addition investigated whether the incorporation of various biomaterials including Matrigel (MA), can augment the differentiation capacity of iMPCs. As the next step, we utilized single-cell RNA-sequencing (scRNA-seq) to dissect cell populations and lineage trajectories indicative of a myogenic regeneration program within constructs. Furthermore, we subjected the constructs to serial cardiotoxin (CTX) injury assay and assessed the potential of iMPCs to regrow aligned myotubes following insult, similiar to regeneration in vivo. Last, to assess the translational applicability of our system, we set out to establish SMCs utilizing human myoblasts that have been subjected to F/R/C supplementation, investigating whether this treatment can enhance their differentiation into aligned myotubes.

## 2 Results

### 2.1 Engineering and characterizing iMPC-derived skeletal muscle constructs (iSMCs)

**Figure 1:**
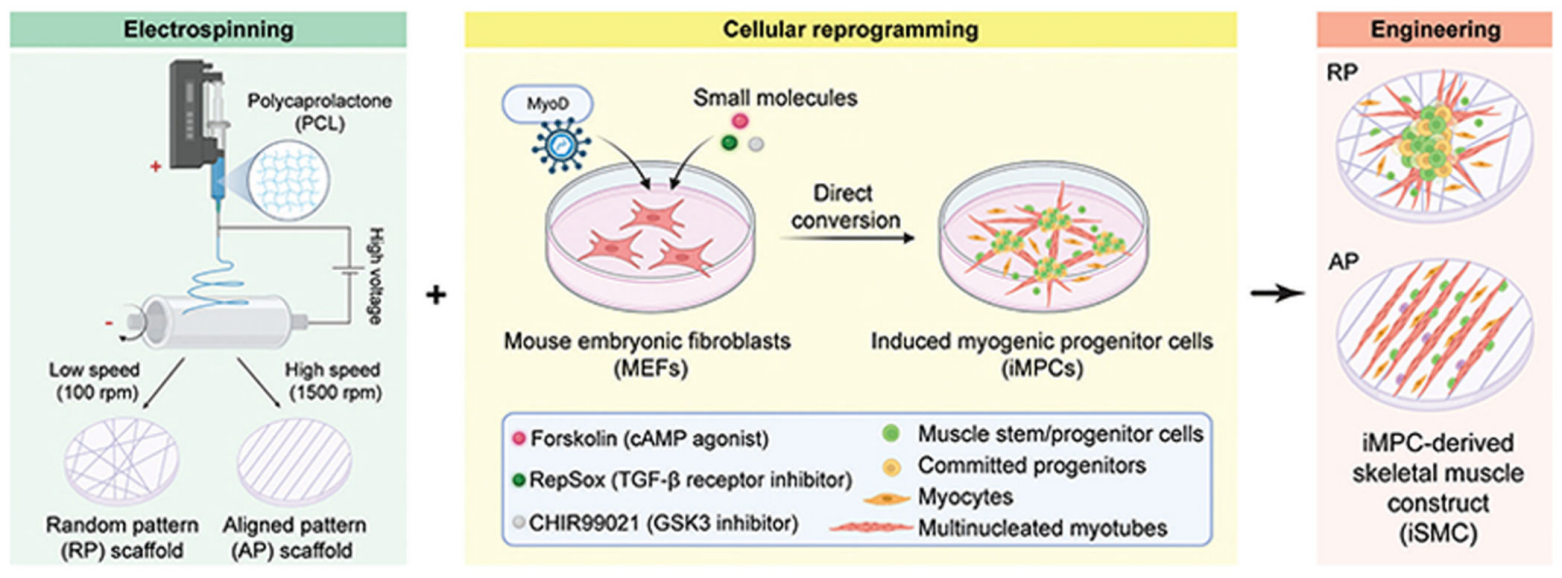
A schematic illustration of experimental design. The schematic illustrates the study objective: engineering skeletal muscle constructs by seeding fibroblast-derived iMPCs onto electrospun PCL scaffolds containing either RP or AP nanofibers.

We set out to generate biomimetic skeletal muscle constructs consisting of aligned myotubes and muscle stem cells in scaffolds, fabricated via electrospinning of PCL nanofibers (Figure 1). By controlling the speed of the rotating collector during electrospinning, two types of electrospun PCL scaffolds were obtained: a random pattern (RP) at low speed (100 rpm) and an aligned pattern (AP) at high speed (1500 rpm) (Figure 1). As the next step, we seeded onto the scaffolds previously generated murine fibroblast-derived iMPCs in the presence of medium containing F/R/C to promote cell expansion and differentiation, aiming to engineer aligned iSMCs containing stem cells, thus resembling a regenerative skeletal muscle tissue (Figure 1).^[42]^

**Figure 2:**
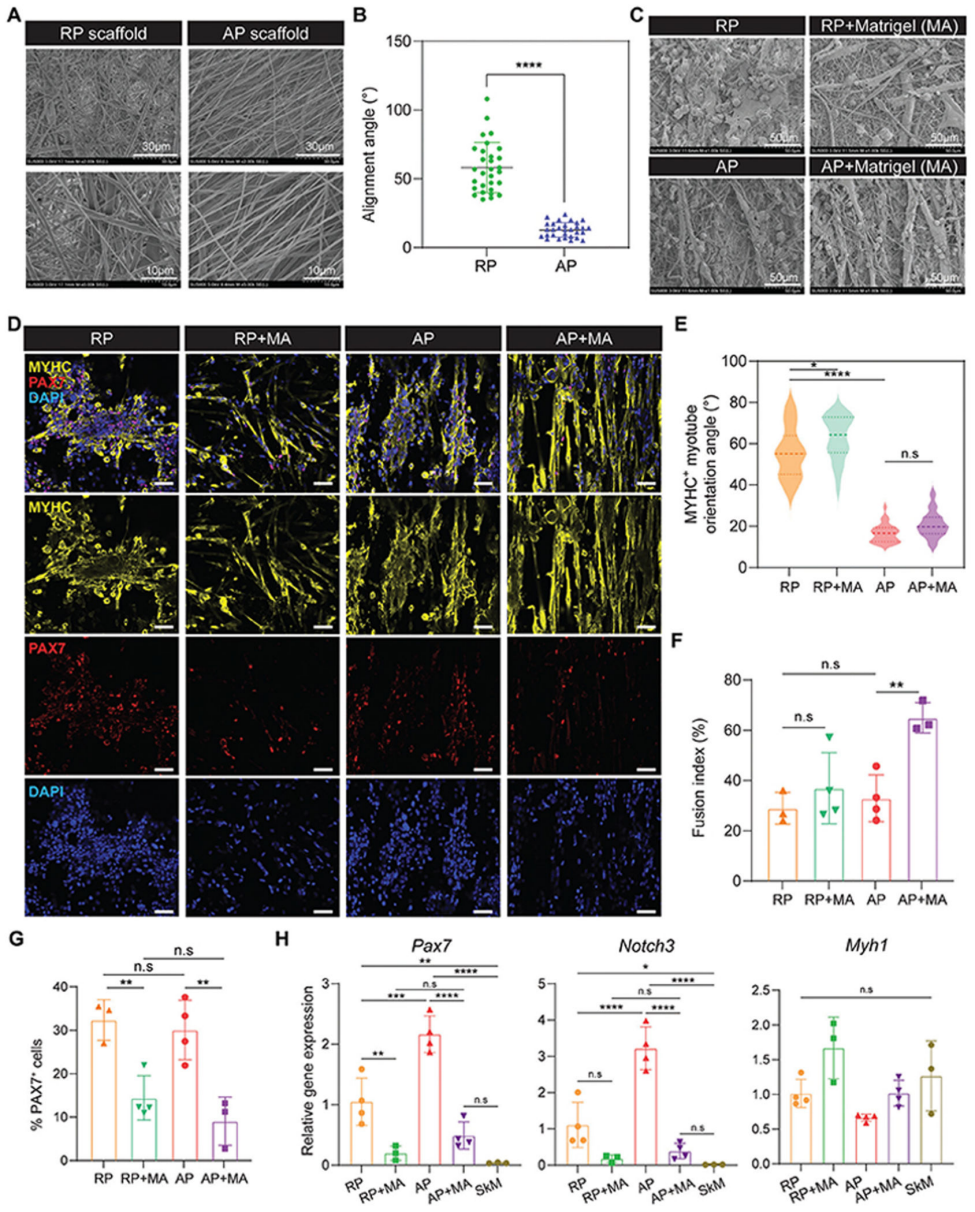
Generation and molecular characterization of iSMCs. A) Electron microscopy images of electrospun PCL scaffolds showing either an aligned or non-aligned nanofibers. Images were acquired by FE-SEM. B) Angle measurement of PCL electrospun fibers shown in (A). The data are shown as means ± S.D (*n* = 30). Statistical significance was determined by a two-tailed unpaired *t*-test (****p < 0.0001). C) FE-SEM images of iMPCs seeded onto electrospun PCL scaffolds in the indicated conditions at day 2 after cell seeding. D) Representative immunofluorescence images of iSMCs stained for MYHC (yellow), PAX7 (red), and DAPI (blue) for the respective conditions. Scale bar, 50 μm. E) A violin plot showing the alignment angle degree of representative MYHC positive myotubes as shown in (D) (*n* = 30). Please note that images with the same field of view at lower magnification are provided in Figure S1C, Supporting information. Statistical significance was determined by one-way ANOVA (*p < 0.05, ****p < 0.0001, n.s = nonsignificant). F) A graph showing fusion index of myotubes, calculated as the percentage of myonuclei in multinucleated myotubes with more than 2 nuclei over all nuclei in an examined field. The data are shown as means ± S.D (*n* = 3–4 independent experiments). Statistical significance was determined by oneway ANOVA (**p < 0.01, n.s = nonsignificant). G) A graph showing the quantification of the percentage of PAX7 positive cells over the total number of DAPI positive cells in iSMCs under the conditions demonstrated in (D). The data are shown as means ± S.D (*n* = 4 independent experiments). Statistical significance was determined by one-way ANOVA (**p < 0.01, n.s = nonsignificant). H) Quantitative RT-PCR of the indicated myogenic stem and differentiation markers. The data are shown as means ± S.D (*n* = 3–4 independent experiments). Statistical significance was determined by one-way ANOVA (*p < 0.05, **p < 0.01, ***p < 0.001, ****p < 0.0001, n.s = nonsignificant). SkM, Skeletal muscle tissue derived from TA muscles.

We first confirmed by Field Emission-Scanning Electron Microscopy (FE-SEM) the unidirectional pattern of the PCL nanofibers in AP scaffolds, whereas a disorganized and non-aligned pattern was formed in RP scaffolds (Figure 2A). To quantify the alignment degree of the nanofibers, we measured their angle orientation and observed significant differences between RP and AP scaffolds (58.2 ± 18.2° and 12.8 ± 5.3° from the point of origin, respectively) (Figures 2A and 2B). Of note, the diameter of electrospun nanofibers in the AP scaffold was smaller due to increased tension elicited by high-speed electrospinning (Figure 2A, Figure S1A,B, Supporting Information). Next, to facilitate a non-toxic environment prior to cell seeding, the scaffolds were treated with Sodium Hydroxide (NaOH) for 4 h, followed by an overnight incubation with 70% ethanol and sterilization by UV light for 30 min. The NaOH treatment was used to increase scaffold hydrophilicity by hydrating ester bonds in the PCL polymers, thereby exposing the carboxyl groups on the surface.^[45]^ In addition, MA was incorporated into a few scaffolds to increase potential biocompatibility and assess the growth of iMPCs when cultured in the presence of potent ECM proteins. This combined effort revealed that under all 4 conditions (RP, AP, RP+MA and AP+MA), a biocompatible environment for iMPC growth was established as observed by FE-SEM (Figure 2C). Remarkably, iMPCs seeded onto AP and AP+MA scaffolds consisted of highly aligned myotubes, which grew in a unidirectional pattern dictated by the orientation of the PCL nanofibers, whereas iMPCs cultured on RP and RP+MA scaffolds exhibited a disorganized orientation pattern of myotubes (Figure 2C).

To molecularly characterize iSMCs, we stained the constructs for the differentiation marker myosin heavy chain (MyHC) and the muscle stem cell marker Pax7, documenting positive cells for either protein under all conditions (Figure 2D and Figure S1C, Supporting Information). In agreement with the FE-SEM analysis, MYHC^+^ myotubes detected under the AP and AP+MA conditions were well-aligned, exhibiting smaller alignment angle in comparison with either the RP or RP+MA conditions (Figure 2D,E and Figure S1C, Supporting Information). Notably, MYHC^+^ myotubes generated under AP+MA displayed a significantly higher fusion index in respect to AP, suggesting elevated differentiation capacity of iMPCs into myotubes in the presence of MA (Figure 2F). Consequently, under the RP and AP conditions, a higher number of PAX7^+^ cells were recorded than under the respective conditions containing MA (≈30% vs 10%) (Figure 2D,G and Figure S1C, Supporting Information). This result was further confirmed by elevated gene expression of other SC markers (*Notch3, Heyl*) under the RP and AP conditions in comparison to the respective MA-supplemented groups (Figure 2H and Figure S1D, Supporting Information). However, the expression of several commitment myogenic genes (*Myod1, Myog*) was not significantly different between the various conditions (Figure S1D, Supporting Information). In addition, we compared the gene expression of iSMCs to that of in vivo *Tibialis anterior* (TA) muscles, and observed that the RP and AP conditions expressed significantly higher levels of SC-related genes than the TA muscle, but a similar level of *Myh1*, most likely due to the low number of SCs present in skeletal muscle tissue, which contains multiple non-myogenic cell types (Figure 2H and Figure S1D, Supporting Information).^[46]^ Taken together, aligned iSMCs were generated by growing iMPCs in biofabricated PCL scaffolds. These constructs consisted of PAX7^+^ stem cells as well as aligned MYHC^+^ myotubes, whose differentiation capacity was augmented by MA supplementation.

### 2.2 Dissecting Proliferation Dynamics and Myogenic Cell Populations in iSMCs

**Figure 3:**
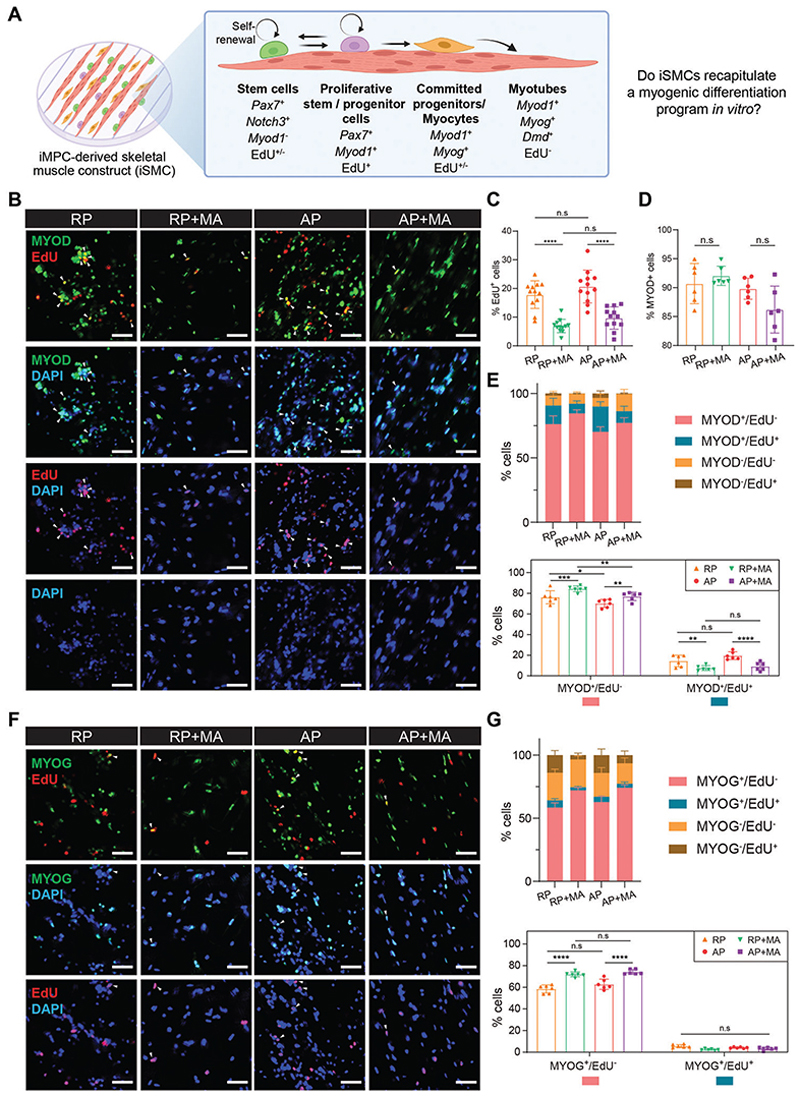
Proliferation and myogenic differentiation analysis of iSMCs. A) An illustration of putative cell populations present in bioengineered iSMCs. B) Representative immunofluorescence images of iSMCs stained for MYOD (green), EdU (red), and DAPI (blue) for the indicated conditions. White arrowheads indicate MYOD^+^/EdU^+^ cells. Scale bar, 50 μm. C) A graph showing quantification of EdU positive cells. The data are shown as means ± S.D (*n* = 12, 3 different images from 4 independent experiments were quantified). Statistical significance was determined by one-way ANOVA (****p < 0.0001, n.s = nonsignificant). D) Quantification of MYOD positive cells shown in (B). The data are shown as means ± S.D (*n* = 6, 3 different images from 2 independent experiments were quantified). Statistical significance was determined by one-way ANOVA (n.s = nonsignificant). E) Quantification of cells subjected to the indicated conditions shown in (B). The data are shown as means ± S.D (*n* = 6, 3 different images from 2 independent experiments were quantified). Statistical significance was determined by two-way ANOVA (*p < 0.05, **p < 0.01, ***p < 0.001, ****p < 0.0001, n.s = nonsignificant). F) Representative immunofluorescence images of iSMCs stained for MYOG (green), EdU (red), and DAPI (blue) for the indicated conditions. White arrowheads indicate MYOG^+^/EdU^+^ cells. Scale bar, 50 μm. G) A graph showing quantification of cells subjected to the indicated conditions shown in (F). The data are shown as means ± S.D (*n* = 6, 3 different images from 2 independent experiments were quantified). Statistical significance was determined by two-way ANOVA (****p < 0.0001, n.s = nonsignificant).

Skeletal muscle differentiation initiates by SCs that undergo activation into proliferative progenitors termed myoblasts and fusion-competent myocytes.^[2]^ Given the presence of both multinucleated MyHC^+^ myotubes and Pax7^+^ stem / progenitor cells in iSMCs, we next opted to characterize the proliferation dynamics and differentiation capacities of myogenic cell populations in iSMCs (Figure 3A). To this end, we performed a 5-Ethynyl-2′-deoxyuridine (EdU) analysis in concert with immunostaining for MyoD, a myoblast and differentiation marker, at day 2 following cell seeding (Figure 3B). In accordance with the PAX7 immunostaining, this analysis revealed a higher percentage of EdU^+^ cells under the RP and AP conditions (17.9 ± 4.8% and 20.7 ± 5.7%, respectively) in comparison to the RP+MA and AP+MA conditions (6.9 ± 2.3% and 9.7 ± 3.9%, respectively) (Figure 3B,C). This observation further supports the notion that MA supplementation elicits cell cycle arrest and differentiation into post-mitotic cells at an early time point during construct development. Of note, among the 4 conditions there were no major differences in the number of MYOD^+^ cells, as in all iSMCs between 80% and 90% MYOD^+^ cells were detected (Figure 3B,D). Further investigation revealed that under these 4 conditions, ≈70% of the cells were MYOD^+^/EdU^−^ and as such non-cycling cells, whereas between 7% and 15% were MYOD^+^/EdU^+^ cycling cells (Figure 3E and Figure S2A, Supporting Information). Interestingly, under RP and AP conditions, a higher percentage of MYOD^+^/EdU^+^ cells and lower MYOD^+^/EdU^−^ cells were detected in comparison to their respective MA-treated conditions, suggesting increased proliferation (Figure 3E and Figure S2A, Supporting Information).

To further corroborate this analysis, we performed immunostaining for the differentiation marker Myogenin (Myog) in concert with an EdU analysis (Figure 3F). In agreement with the immunostaining for MYOD, iSMCs under all conditions had ≈60%–70% MYOG^+^/EdU^−^ differentiated cells, while MA incorporation increased the number of non-proliferating MYOG^+^ cells compared to the respective condition without MA (Figure 3G and Figure S2B, Supporting Information). Of note, a small percentage of MYOG^+^/EdU^+^ cells were detected across all conditions, in accordance with a previous study that reported proliferation of Myog-expressing mononucleated cells (Figure 3G and Figure S2C, Supporting Information).^[47]^ In summary, our investigation revealed several myogenic cell populations in iSMCs under all conditions, without major differences between aligned or randomly patterned iSMCs. However, fewer proliferating cells were detected in the presence of MA, suggesting enhanced terminal differentiation when using this biomaterial. This observation is in agreement with the higher fusion index documented in MA-treated iSMCs, as well as the reduction in the number of PAX7^+^ cells (Figure 2).

### 2.3 The Effect of Biomaterials on the Myogenic Potential of iSMCs During Prolonged Culture

**Figure 4:**
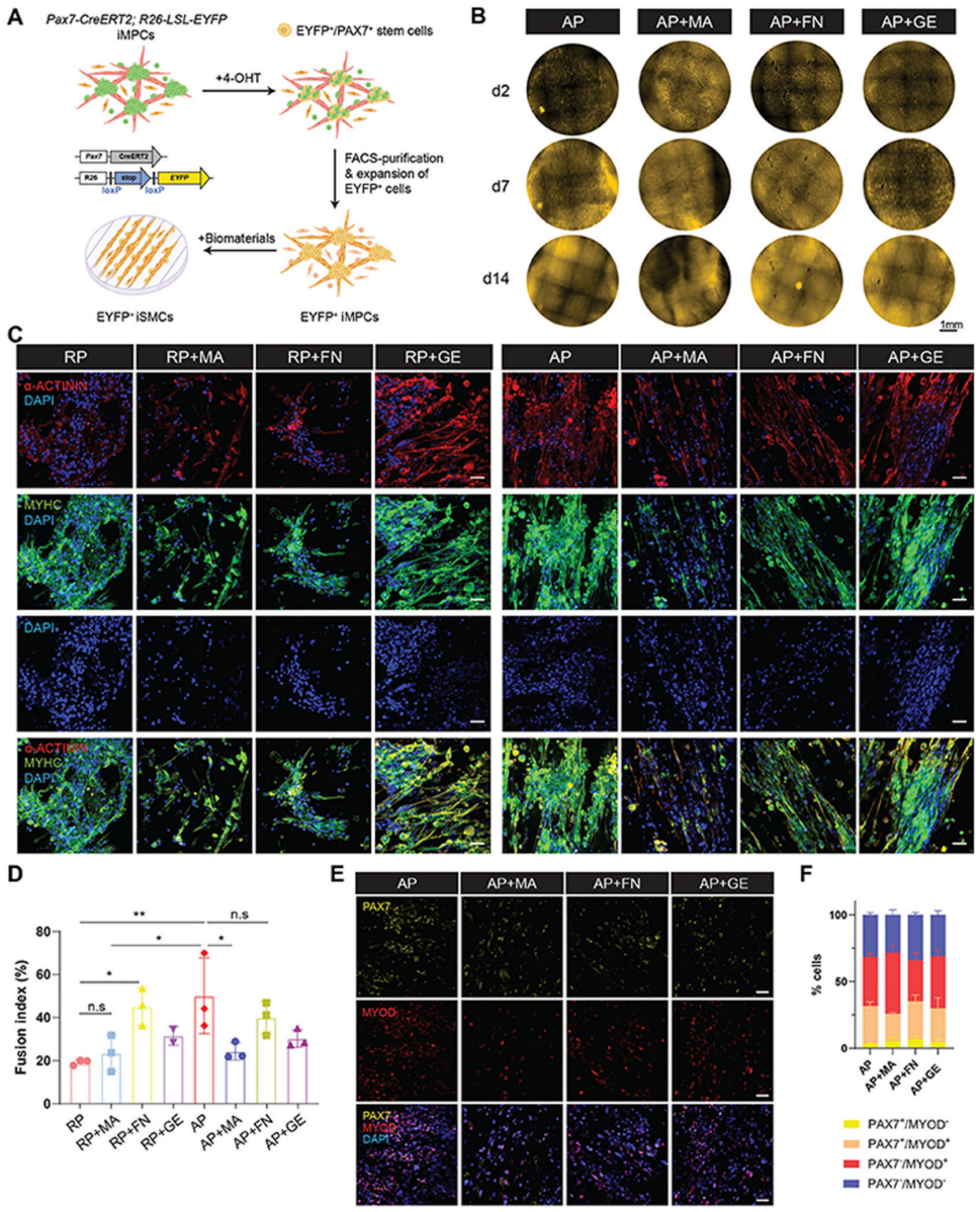
Assessing the effect of biomaterials on the derivation of iSMCs. A) A schematic illustration of the objective to assess the derivation of EYFP^+^ iSMCs in the presence of various biomaterials. B) Fluorescence images of EYFP^+^ iSMCs established with biomaterials at the indicated days. Entire constructs were captured by microscopy, and the center of each construct is displayed. Scale bar, 1 mm. MA, Matrigel, FN, Fibronectin, GE, Gelatin. C) Representative immunofluorescence images of iSMCs stained with sarcomeric 𝛼-ACTININ and MYHC at day 14. MYHC was stained in the Cy3 channel and colored as pseudo-green. Scale bar, 50 μm. D) A graph showing fusion index analysis calculated based on the images shown in (C). The data are shown as means ± S.D (*n* = 3). Statistical significance was determined by one-way ANOVA (*p < 0.05, **p < 0.01, n.s = nonsignificant). E) Representative immunofluorescence images of iSMCs stained with PAX7 and MYOD after 7 days of culture. Scale bar, 50 μm. F) Quantification of (E). The data are shown as means ± S.D (*n* = 3).

Our analyses until this stage were performed at day 2 after the generation of iSMCs. As a next step, we opted to investigate whether iSMCs can survive and expand for a longer time period when cultured in the presence of various biomaterials. To visualize the cells in iSMCs during extended culture, we utilized EYFP^+^ iMPCs generated from *Pax7-CreERT2; Rosa26-LSL-EYFP* fibroblasts.^[35]^ Following 4-Hydroxytamoxifen (4-OHT) administration and fluorescence-activated cell sorting (FACS)-purification, EYFP^+^ iMPCs were seeded onto PCL scaffolds (Figure 4A). Additionally, we incorporated a variety of biomaterials, including MA, Fibronectin (FN) and Gelatin (GE), to examine their effect on the cell growth in iSMCs. Consequently, we observed that the conditions of AP, AP+FN and AP+GE showed extensive cell growth up to 14 days of culture, however the AP+MA condition displayed early differentiation at day 2, and extensive cell loss by day 14 based on EYFP expression (Figure 4B). We attribute this observation to reduction in Pax7^+^ stem cells following MA treatment, which most likely mitigated the proliferative potential of MA-treated iSMCs over time.

To molecularly characterize iSMCs generated using these biomaterials, and in particular investigate their effect on the maturation of iSMCs, we performed immunostaining for 𝛼-ACTININ, an established sarcomeric marker for myotube maturation. We noted that at day 14, most conditions in the AP group demonstrated enhanced formation of 𝛼-ACTININ^+^ striated myotubes in comparison to the RP groups, aside from the AP+MA condition (Figure 4C and Figure S3A, Supporting Information). In addition, the AP condition exhibited a higher fusion index in comparison to the RP, RP+MA and AP+MA conditions (Figures 4C and 4D). Notably, under the AP+FN or RP+FN condition, we noted a similar or a higher fusion index than for the AP or RP conditions, respectively, but not under the GE condition (Figure 4D). Moreover, by performing a co-immunostaining for PAX7 and MYOD, we noted that under the AP+MA condition, relatively less PAX7^+^/MYOD^+^ cells were detected (Figure 4E,F, Figure S3B,C, Supporting Information). These analyses imply that the AP condition, with or without FN and GE supplementation, contain a larger proliferative stem cell pool than the AP+MA condition, thereby enabling to maintain iSMCs for a longer time period as judged by continuous myotube formation during extended culture. Taken together, we successfully developed iSMCs with additional biomaterials whose biochemical composition is more defined, demonstrating their growth potential and capacity to produce mature myotubes with time. We also conclude that MA supplementation enhanced the differentiation of iSMCs at an early time point, however was unfavorable for long-term iSMC maintenance due to a reduction in the number of Pax7 expressing cells.

### 2.4 Deconstructing Cellular Heterogeneity of iSMCs by scRNA-seq

**Figure 5:**
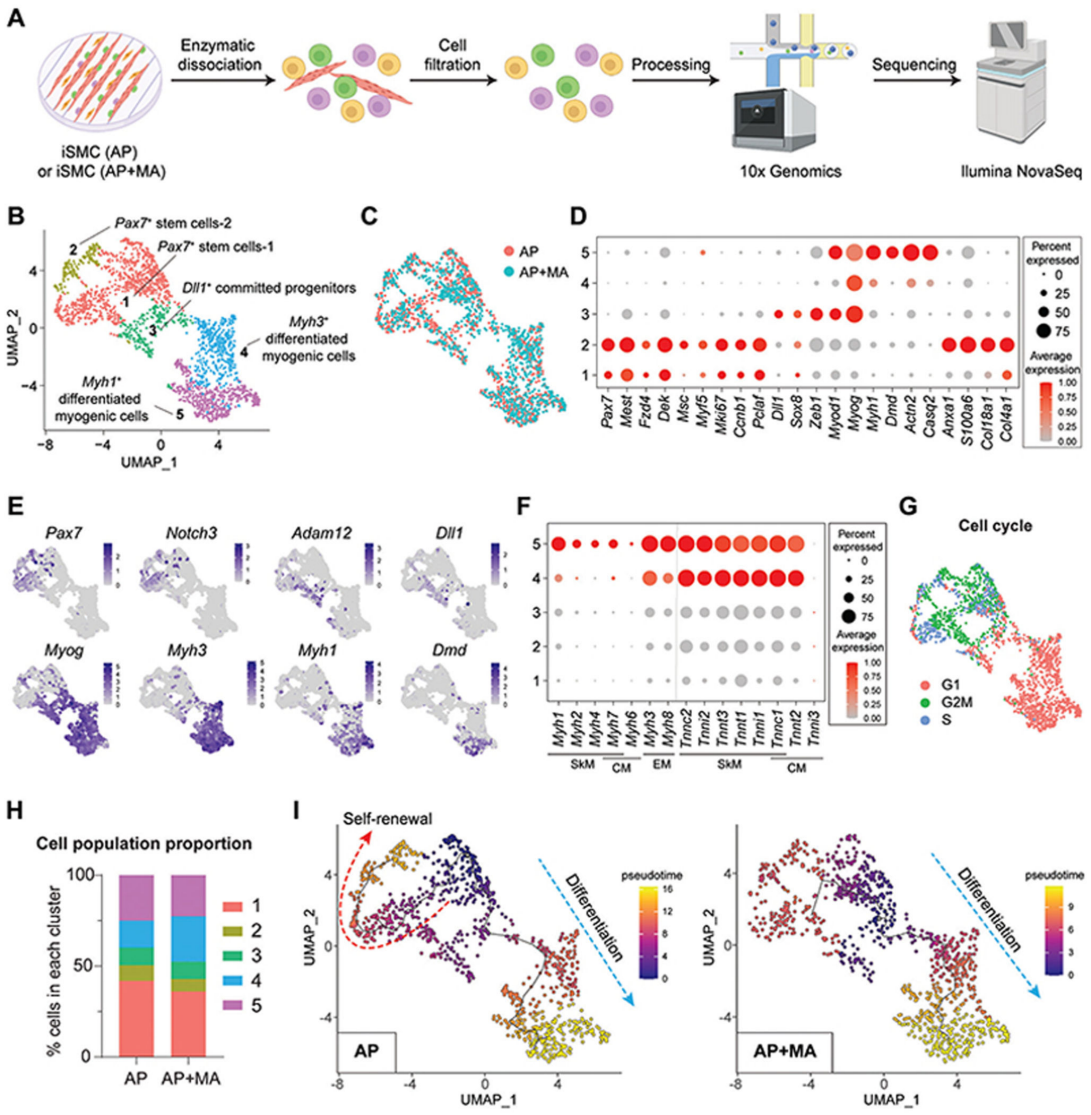
scRNA-seq analysis of iSMCs. A) Graphical schematic of experimental design to analyze by scRNA-seq the cell populations that comprise iSMCs. B) UMAP projection showing the integrated objects of AP and AP+MA conditions, denoting 967 and 989 cells extracted for scRNA-seq analysis of iSMCs under the AP and AP+MA conditions, respectively. Cells are colored by clusters. C) UMAP projection of integrated scRNA datasets colored by condition. D) Dot plot showing the expression level of the indicated marker genes for each cell cluster. E) Feature plots showing the expression level of the indicated genes. F) Dot plot showing the expression level of the indicated genes that are associated with skeletal muscle (SkM), cardiac muscle (CM) and embryonic muscle (EM). G) UMAP projection of integrated scRNA datasets colored by each cell cycle state. H) Bar graph showing the cell cluster distribution in percentages for the indicated iSMCs. I) Single cell pseudotime trajectory analysis of the two indicated iSMCs. Cells are colored by pseudotime values.

Our analyses thus far revealed distinct cell populations in iSMCs consisting of stem, progenitor and differentiated cells. To further characterize the transcriptional heterogeneity of aligned iSMCs as well as the effect of MA supplementation, we conducted scRNA-seq analysis of iSMCs under AP or AP+MA conditions (Figure 5A). First, the cells were collected from iSMCs by enzymatic digestion, and then subjected to 10× platform processing and sequencing after removal of multinucleated myotubes by filtration (Figure 5A). The sequencing data were processed individually using the Seurat v4.2.1 pipeline^[48]^ for each condition, and integrated together as represented by a unified UMAP (Figure 5B). Overall, 5 cell clusters (C) were detected in the integrated UMAP and no difference was observed in cell type composition between the AP and AP+MA conditions (Figure 5B,C and Figure S4A, Supporting Information). These 5 cell clusters were annotated as two stem cell populations that were positive for *Pax7* (C1, C2), a committed progenitor cell population expressing *Dll1* (C3) and two differentiated myogenic cell populations expressing *Myog* (C4, C5) (Figure 5B–E and Figure S4A, Supporting Information).

Next, a closer investigation into the two stem cell clusters C1 and C2 revealed similar expression of SC-associated genes including *Pax7, Msc, Myf5* and *Heyl*: however, C2 further exhibited expression of ECM-related genes including *Col18a1* and *S100a6*, as previously observed (Figure 5D,E, Figure S4B,C, Supporting Information).^[42]^ The committed progenitor cell population (C3) expressed *Dll1, Sox8* and *Zeb1* (Figure 5D,E and Figure S4B, Supporting Information). In a former study, we reported on an intermediate cell cluster in iMPCs that expressed *Sox8* and *Dll1*, which interacted with Notch receptor expressing muscle stem cells via ligand-receptor binding.^[42]^ The detection of this cluster in iSMCs supports our prior assumption that this cell population represents a nexus between myogenic stem and differentiated cells in iMPCs. In respect to the differentiated cell clusters C4 and C5, both expressed *troponin* isoforms and embryonic *Myh* isoforms such as *Myh3* and *Myh8*, yet C5 exhibited a higher expression of terminally differentiated markers including fast- and slow-twitching *Myh* isoforms (*Myh1, Myh4, Myh*7), dystrophin (*Dmd), Actn2* and *Casq2* (Figure 5D–F). This observation implies that C5 lies at the tail end of the myogenic differentiation program, denoting mononucleated cells that have exited the cell cycle prior to cell fusion. In support of this finding, a cell cycle analysis revealed a high expression of cell cycle regulators indicative of a proliferation state (S and G2M) in the stem and progenitor cell populations C1-3, and a high expression of non-proliferating cell cycle regulators (G1) in the differentiated cell populations C4-5 (Figure 5G). Although we did not detect prominent differences between the conditions AP and AP+MA, the relative cell fraction of C1 was slightly higher under the AP condition (41.9% in AP vs 36.0% in AP+MA), whereas that of C4 was higher under the AP+MA condition (14.8% in AP vs 25.0% in AP+MA) (Figure 5H). As C1 is enriched in stem cell markers such as *Pax7, Heyl* and *Fzd4*, whereas C4 is enriched in differentiation markers such as *Myog*, this analysis supports our prior observation that MA supplementation reduces the number of stem cells while augmenting differentiation capacities.

To gain further insights into the cell transition dynamics during myogenic differentiation in iSMCs, we performed a pseudotime trajectory analysis selecting C1 as the root node. This effort revealed that the initiation of the myogenic differentiation program under the AP condition bifurcates into either C2 or C3-5 arm, whereas under AP+MA the initiation similarly starts at C1 however predominantly proceeds toward the C3-5 arm (Figure 5I). This observation cautiously implies an impaired self-renewal in the stem cell populations C1 and C2 under the AP+MA condition. However, a confirmation of this analysis would require further investigation to rule out the possibility that this effect might be derived from technical limitations due to low gene/UMI counts under the AP+MA condition. In summary, using scRNA-seq analysis we unveiled the cell populations comprising iSMCs, dissecting the different stages of the myogenic differentiation program. Overall, no major transcriptional differences were detected between the two examined conditions. However, subtle differences between the AP and AP+MA conditions were observed for certain cell populations, pointing toward an increase in self-renewal of PAX7^+^ stem cells under the AP condition, and an augmented myogenic differentiation propensity under the AP+MA condition.

### 2.5 Assessing the Regeneration Potential of iSMCs in an Injury-Based Model

**Figure 6:**
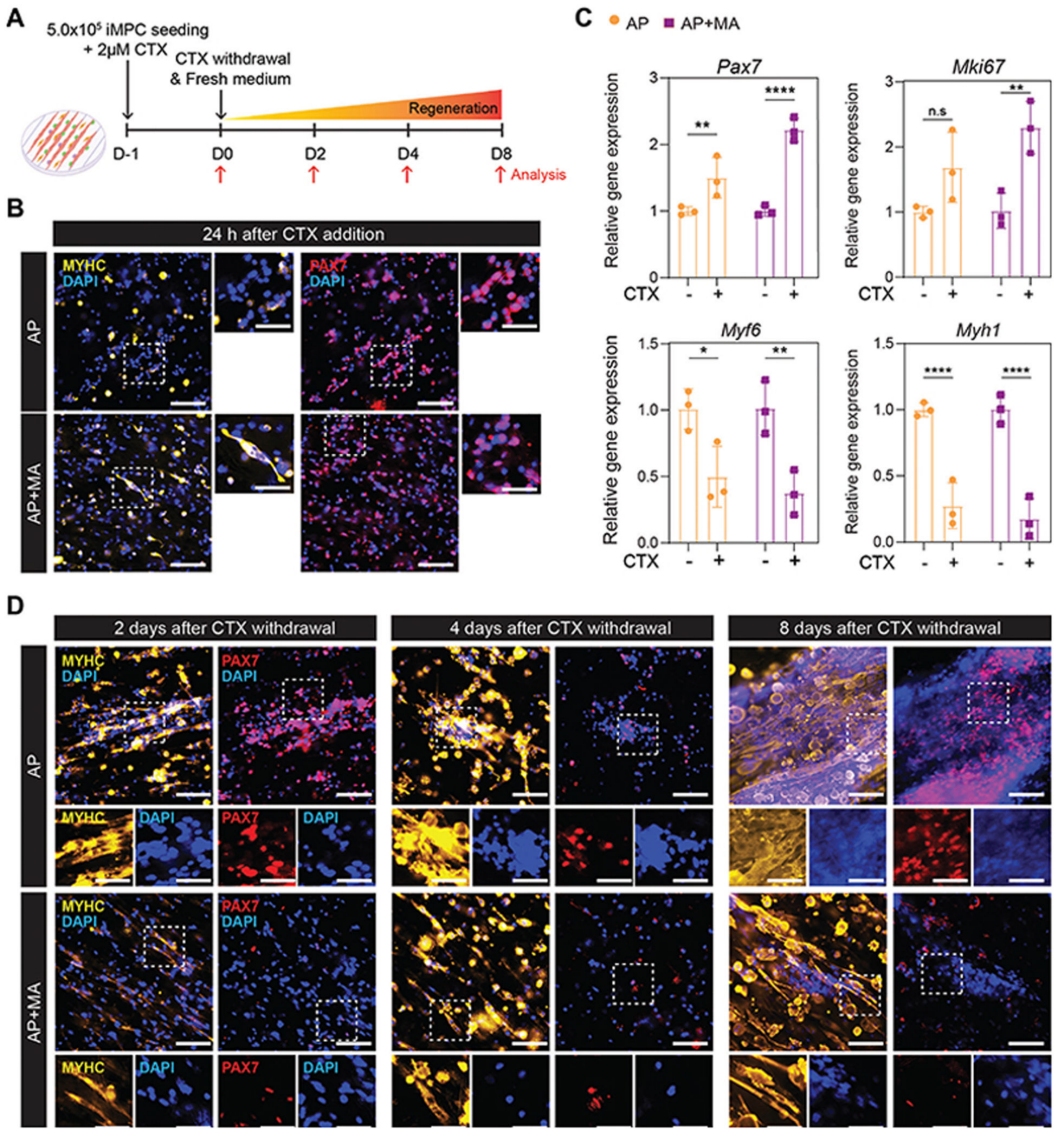
Analysis of iSMCs subjected to a myonecrotic agent. A) Graphical schematic illustrating the indicated time points used to evaluate the regeneration capacity of iSMCs subjected to a CTX-induced myofiber damage. B) Representative immunofluorescence images of iSMCs stained for MYHC (yellow), PAX7 (red), and DAPI (blue) in the respective conditions. The images were taken 1 day after CTX treatment. Scale bar, 100 μm. Scale bar of zoom-in images, 50 μm. C) qRT-PCR analysis of the indicated genes that are associated with a myogenic stem or differentiated cell fate, as well as the proliferation marker *Mki67*. The data are shown as means ± S.D (*n* = 3 independent experiments). Statistical significance was determined by one-way ANOVA (*p < 0.05, **p < 0.01, ****p < 0.0001, n.s = nonsignificant). D) Representative immunofluorescence images of iSMCs stained for MYHC (yellow), PAX7 (red), and DAPI (blue) in the indicated conditions. Scale bar, 100 μm. Scale bar of zoom-in images, 50 μm.

Our results thus far revealed that iSMCs contain proliferating PAX7^+^ stem cells that can efficiently differentiate into aligned myotubes. This observation raised the interesting question whether iMPC-derived biomimetic constructs can regrow aligned myotubes following an ectopic insult, similar to muscle regeneration in vivo. To investigate this hypothesis, we treated iSMCs with CTX, a widely used myonecrotic agent that can trigger extensive damage to muscle fibers but rarely to satellite cells (Figure 6A).^[49,50]^ We chose to analyze iSMCs across various time points upon CTX administration during cell seeding, as well as after CTX withdrawal (Figure 6A). At 24 h post CTX administration, a few MYHC^+^ myotubes were detected under either the AP or AP+MA conditions, although many PAX7^+^ cells were observed (Figure 6B). Accordingly, we noted that CTX administration increased the expression level of *Pax7* and the proliferation marker *Mki67*, whereas it decreased the expression level of differentiation related genes such as *Myf6* and *Myh1* under both conditions, most likely due to the depletion of differentiated skeletal muscle cells by CTX (Figure 6C). Moreover, we observed that CTX treatment did not affect the viability of PAX7^+^ cells as recorded by Calcein-AM staining of *Pax7-CreERT2; R26-LSL-ntdTomato* iMPCs labeled with 4-OHT (Figure S5A–C, Supporting Information). At 2 days post CTX administration, we analyzed the constructs and observed that multinucleated MYHC^+^ myotubes were predominantly formed under the AP condition, growing in size at days 4 and 8 (Figure 6D). Accordingly, we observed more PAX7^+^ cells under the AP condition at the analyzed time points (Figure 6D). At day 8 post CTX administration, large multinucleated myotubes were detected under the AP condition, in concert with increased number of PAX7^+^ stem cells (Figure 6D).

**Figure 7:**
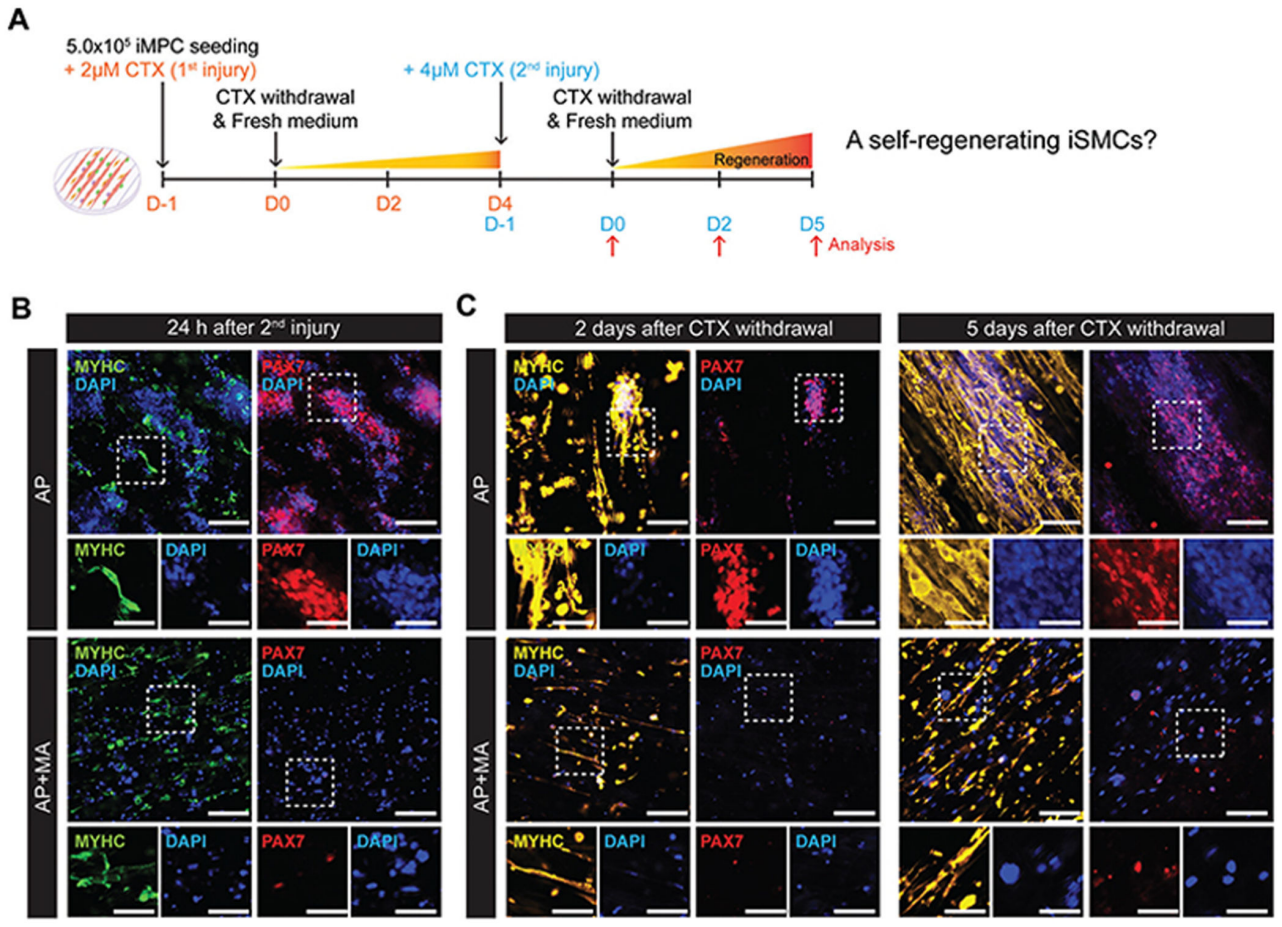
Analysis of iSMCs subjected to a serial injury assay. A) Graphical schematic illustrating the experimental procedure used to assess the regeneration capacity of iSMCs in a re-injury CTX model. B) Representative immunofluorescence images of iSMCs stained for MYHC (green), PAX7 (red), and DAPI (blue) in the respective indicated conditions. The images were taken 1 day after the second CTX treatment. MYHC was stained in the Cy3 channel and colored as pseudo-green. Scale bar, 100 μm. Scale bar of zoom-in images, 50 μm. C) Representative immunofluorescence images of iSMCs undergoing regeneration after a second CTX injury and stained for MYHC (yellow), PAX7 (red), and DAPI (blue) for the indicated conditions. The images were taken 2 or 5 days after CTX withdrawal. Scale bar, 100 μm. Scale bar of zoom-in images, 50 μm.

Given the abundancy of PAX7^+^ stem cells detected after CTX exposure in the AP condition, we subjected iSMCs to a second CTX administration to assess their regeneration competency in a serial injury model. Under this experimental design, the second CTX treatment was higher in concentration and applied to the iSMCs 4 days after recovery from the first CTX treatment, and the respective analysis was performed up to 5 days after the second injury (Figure 7A). Similar to the first CTX treatment, another round of injury resulted in extensive damage to myotubes under both conditions (Figure 7B). Importantly, the AP+MA condition poorly regenerated myotubes after a second CTX treatment, whereas the AP condition successfully regenerated aligned iSMCs consisting of PAX7^+^ stem cells and MYHC^+^ myotubes 5 days after reinjury (Figure 7C). This observation suggests that self-renewing PAX7^+^ stem cells are important for regenerating myotubes upon CTX-induced muscle damage. These results are also in line with the scRNA-seq analysis, which demonstrated that a cycling PAX7^+^ stem cell population gave rise to differentiated muscle cells in iSMCs. In summary, following a single or a serial CTX injury, self-renewing muscle stem cells were able to regenerate aligned myotubes in iSMCs, resembling myofiber regeneration in vivo.

### 2.6 Establishing Human Skeletal Myoblast-Derived SMCs with Small Molecule Treatment

**Figure 8:**
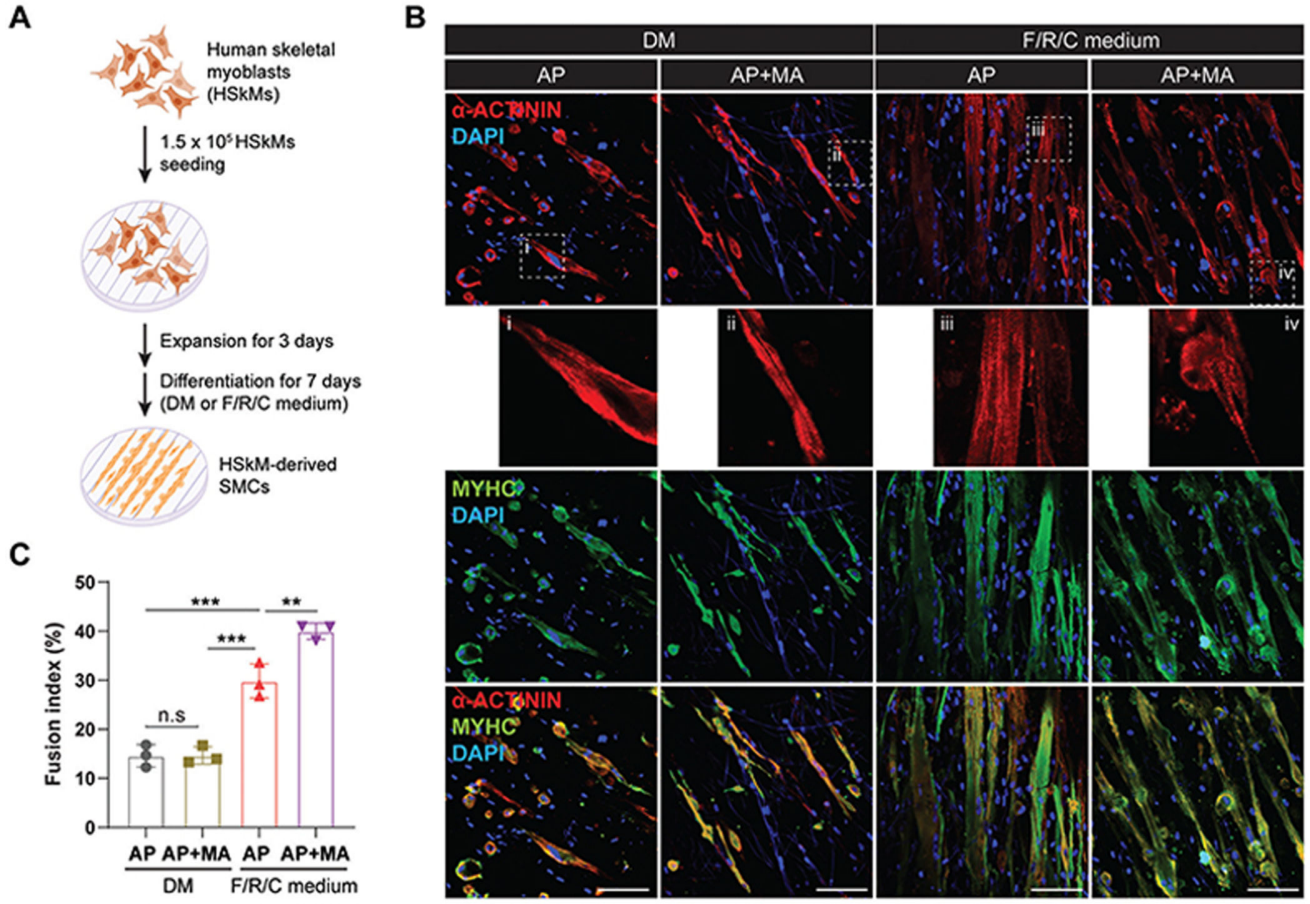
Derivation of human myoblast-derived SMCs with small molecule treatment. A) An experimental design to produce hSMCs with F/R/C treatment. DM, Differentiation medium. B) Representative immunofluorescence images of hSMCs stained for the indicated markers. Scale bar, 100 μm. C) A graph showing fusion index analysis based on (B). The data are shown as means ± S.D (*n* = 3). Statistical significance was determined by one-way ANOVA (**p < 0.01, ***p < 0.001, n.s = nonsignificant).

Our results thus far documented production of iSMCs using murine fibroblast-derived iMPCs. However, whether human skeletal muscle cells, which represent a more therapeutically-relevant model than mouse cells, can respond to F/R/C treatment and give rise to highly differentiated SMCs is unknown. To address this question, we opted to employ human skeletal myoblasts (HSkMs) to produce human SMCs (hSMCs) under the AP or AP+MA conditions. To this end, HSkMs were seeded onto electrospun scaffolds, cultured in growth medium for 3 days and then differentiated either with a conventional differentiation medium (DM) or F/R/C medium for 7 days prior to analysis (Figure 8A). Under both conditions, we were able to detect aligned myotubes, however remarkably in comparison to the DM treatment, hSMCs subjected to F/R/C treatment exhibited increased differentiation capacity, as judged by the presence of MYHC^+^/𝛼-ACTININ^+^ striated myotubes (Figure 8B). Accordingly, the fusion index of hSMCs treated with F/R/C was significantly higher than that of DM-derived hSMCs (Figure 8C). Moreover, in accordance with our prior results, MA supplementation increased the fusion index of F/R/C-derived hSMCs, however surprisingly not of DM-derived hSMCs (Figure 8C). Collectively, these results demonstrate that F/R/C supplementation can facilitate myogenesis and increase differentiation capacities and maturation of human myoblasts seeded onto electrospun substrates, raising the possibility of using this system for translational applications.

## 3 Discussion

In this study, we established a biomimetic skeletal muscle construct by seeding iMPCs on electrospun PCL substrates, resulting in myotube alignment in conjunction with self-renewing muscle stem cells. Furthermore, incorporation of MA enhanced the differentiation capacity of iMPCs in the scaffolds, while mitigating their self-renewal, thus rendering long-term MA treatment unfavorable for maintenance of iSMCs. In addition, we dissected cell populations comprising iSMCs using a variety of molecular assays including scRNA-seq, and demonstrated their capacity to regenerate aligned myotubes in a single or serial injury model. Last, we documented that small molecule treatment proved beneficial for production of highly differentiated and aligned hSMCs.

Several implications extend from our study. In respect to the use of MA, we envision it entails both advantages and disadvantages for tissue engineering purposes. MA is an intricate and undefined macromolecule protein matrix that is widely used for conventional myoblast culture, as it enables SC attachment to plastic dishes and supports robust proliferation in medium containing high serum.^[8,51]^ However, for the system reported in this study, MA induced morphological and cellular changes in iSMCs, manifesting increased differentiation and mitigating self-renewal of muscle stem cells after a few days of construct development. We hypothesize this is most likely due to signaling molecules present in MA such as IGF1, EGF and FGF2,^[52–54]^ which were reported to play an important role in regulating SC activation and muscle regeneration.^[55–58]^ Moreover, the composition of MA is rather similar to that of the ECM of skeletal muscle tissue, which is known to regulate stem cell activation.^[59]^ In particular, laminin, a major component of MA, is abundant in binding sites for integrin 𝛼7, a protein known to play a key role in SC activation and fusion.^[57–60]^

Overall, we observed that MA supplementation rapidly enhanced myotube formation, however long-term exposure to MA depleted the stem cell pool in iSMCs, rendering other biomaterials such as FN and GE more favorable for long-term maintenance. We attribute this observation to the different biomaterial composition which may affect cellular differentiation pathways. For example, MA is a macromolecular matrix consisting of laminin, collagen IV, and other proteoglycans,^[61]^ whereas FN and GE contain a more simplified composition including a protein dimer linked by disulfide bonds and a protein-based polymer produced by hydrolysis of collagen, respectively. This simplified composition may be less favorable for promoting differentiation of muscle stem cells, thereby enabling their proliferation in iSMCs for a longer time period. Additionally and notably, we observed by a scRNA-seq analysis that MA’s effect was rather subtle, primarily affecting two cell populations in iSMCs, including stem and differentiated cell clusters. It is important to note, however, that scRNA-seq captures only the mononucleated fraction of iSMCs, and as such conducting single nucleus RNA-seq analysis of multinucleated myotubes generated with or without MA might lend additional insights into the potential effect of MA supplementation on the maturation state of iSMCs. Last, an alternative explanation to the effect of biochemical cues on iSMC maturation could entail biophysical cues. However, the elastic modulus of electrospun PCL substrates is ≈20–200 MPa, whereas the elastic modulus of MA, or other biomaterials is less than 1 kPa^[62]^ therefore their impact on the elastic modulus of PCL substrates is most likely negligible.

To date, a multitude of studies reported aligned myotube formation in 3D for tissue engineering purposes.^[9–11,31]^ In respect to the method used in this study, the advantages for using PCL is its biocompatibility, easy modification and FDA-approval.^[63]^ Additionally, PCL does not include cell adhesion proteins that might affect the interactions between the cell populations comprising iMPCs, which are highly heterogeneous and require expansion in the presence of a small molecule cocktail that modulates several signaling pathways.^[35]^ Given that PCL is also biodegradable, the use of iSMCs is attractive for in vivo therapeutic approaches aiming to treat extensive muscle degeneration in the form of latestage Duchenne muscular dystrophy (DMD) or volumetric muscle loss (VML).^[64]^

Another advantage of the approach reported herein is the unique use of iMPCs as a cell source to engineer skeletal muscle. As of today, only one study reported using directly reprogrammed myogenic progenitors for tissue engineering purposes, showcasing their potential to treat VML.^[64]^ Similarly, we believe the use of iMPCs was key to rapidly producing iSMCs, as solely seeding iMPCs in the presence of high serum and F/R/C supplementation for as little as 2 days was required. This is a consequence of the intrinsic attributes of iMPCs, including rapid muscle stem cell proliferation in conjunction with robust differentiation into myotubes, unlike most conventional myoblast cell lines that are cultured either under proliferation or differentiation conditions.^[8,42]^ For instance, the most widely used immortalized myogenic cell line C2C12 is typically employed only for its fusion attributes.^[65]^ However, C2C12 is karyotypically abnormal, immortalized and lacks primary satellite-like muscle stem cells, representing a major limitation.^[7,65,66]^

In comparison to iMPCs cultured on 2D plates, iSMCs form a more organized model, yet they do not fully mirror an in vivo skeletal muscle tissue which contains a variety of cell types including SCs, that are quiescent under homeostatic conditions.^[46]^ In contrast, iSMCs appear highly proliferative, mirroring an ongoing regeneration response in vitro, most likely due to the effect of the F/R/C small molecule cocktail on SC activation. It will be of interest to assess whether a few Pax7^+^ cells in iSMCs exit the cell cycle and return to quiescence, as previously reported for SCs seeded on myofibers.^[67–69]^ Additionally, it will be of interest to assess whether serum withdrawal or modulation of additional signaling pathways might confer quiescence rather than activation on the Pax7^+^ cell population in iSMCs, rendering them akin to muscle tissue during homeostasis.^[68,70,71]^

Constructing an intact skeletal muscle tissue in vitro is a major challenge, as in addition to SCs and muscle fibers skeletal muscle tissue contains a plethora of additional resident cells including endothelial cells, fibro-adipogenic progenitors, immune cells and motor neurons.^[46]^ It will be of interest to seed such cell types into iSMCs to generate a more physiologically relevant construct that mimics cellular attributes and interactions present in muscle tissue in vivo. Furthermore, we did not detect robust spontaneous contractions in iSMCs, whereas iMPCs sometimes contract in 2D culture plates. As such, it will be of interest to investigate in the future this functional electrophysiological property by actively inducing contractions in iSMCs via optogenetic, chemical or electrical stimuli as previously shown for other myogenic cell types in 3D.^[32,50,72]^ Finally, an attractive utility for iSMCs may involve modeling muscular dystrophies such as DMD in vitro, potentially replacing animal models and supporting the “Replace, Reduce, Refine” (3R) animal experimentation guidelines. To date, several studies reported on fabricating 3D engineered skeletal muscle constructs using DMD patient-derived myoblasts^[73]^ or iPSC-derived myogenic precursor cells.^[28,74]^ Our effort to harness F/R/C treatment to produce human constructs with augmented differentiation capacity adds to the milieu of such important endeavors, and proposes an alternative method to derive patient-specific myogenic constructs. Respectively, our lab recently developed an in vitro cellular disease model for DMD by reprogramming dystrophic mouse fibroblasts into iMPCs that did not express the dystrophin protein, enabling to assess dystrophin restoration by CRISPR-Cas9.^[75]^ We envision that derivation of mouse iSMCs or human SMCs from DMD myogenic cells may provide an optimized in vitro model for basic research applications, or as a platform to test therapeutic interventions that may prove beneficial for DMD patients.

## 4 Experimental Section

### Fabrication of PCL Scaffolds Using Electrospinning

To produce electrospun PCL scaffolds, PCL (Sigma-Aldrich, 440 744, Mn = 80,000 g mol^−1^) that was dissolved for 24 h under constant stirring at 10% (w/v) in a 4:1 chloroform: ethanol mixture was used. Electrospinning was performed using a commercially available electrospinning machine (IME Technologies, EC-CLI) for 60 min using the following parameters and conditions: electric potential of the nozzle = 15 kV, electric potential of the collector = −2 kV, size of needle = 22 G, flow rate = 7.5 mL h^−1^, distance between nozzle and collector = 15 cm and mandrel rotation speed = 100 or 1500 RPM depending on the scaffold group. Electrospun nanofibers were collected onto a Ø 90 × 180 mm cylindrical mandrel at room temperature (RT). After spinning, the membranes were cut into cylinders with a Ø 8 mm biopsy punch.

### Surface Modification and Sterilization of PCL Scaffolds

PCL membranes were treated with 1 m NaOH for 4 h, followed by several washes with PBS and sterilization in 70% Ethanol overnight. The scaffolds were then washed with PBS in sterile conditions, placed under UV lamp for 20 min and used for cell seeding.

### Fiber Alignment Angle and Fiber Size Measurement

As a first step, the fibrous structure images comprising the electrospun scaffolds were captured by FE-SEM. Next, acquired images were analyzed using the ImageJ software (NIH, United States) to measure the alignment angle and fiber diameter. The alignment angle of electrospun fibers was measured by using the “Angle Tool” function in ImageJ which measures the angles between the pattern direction and the longest axis of the fiber. For the fiber diameter measurement, the “Set Scale” function in ImageJ was used first to set unit length by converting the obtained length from the scale bar in the FE-SEM image. Then, a “Straight line” function in ImageJ was used to measure the fiber diameter from the image.

### Cell Media Composition

The F/R/C medium contained KnockOut (KO) DMEM (Thermo Fisher Scientific, 10 829 018) supplemented with 10% KO Serum Replacement (Thermo Fisher Scientific, 10 828 028), 10% FBS (Thermo Fisher Scientific, 10 270 106), 1% GlutaMAX (Thermo Fisher Scientific, 35 050 061), 1% non-essential amino acids (Thermo Fisher Scientific, 11 140 050), 1% Pen-Strep (Thermo Fisher Scientific, 15 140 122), 0.1% 2-Mercaptoethanol (Thermo Fisher Scientific, 21 985 023), 10 ng mL^−1^ basic FGF (R&D Systems, 233-FB), 5 μm Forskolin (R&D Systems, 1099/50), 5 μm RepSox (R&D Systems, 3742/50), and 3 μm CHIR99021 (R&D Systems, 4423/50). The growth medium of HSkMs contained 40% Ham’s F-10 Nutrient mix (Thermo Fisher Scientific, 22 390 025), 40% low glucose DMEM (Thermo Fisher Scientific, 31 885 023), 20% FBS, 1% Pen-Strep and 10 ng mL^−1^ basic FGF. The differentiation medium of HSkMs contained high glucose DMEM (Thermo Fisher Scientific, 41 966 029) supplemented with 2% horse serum (Thermo Fisher Scientific, 16 050 122) and 1% Pen-Strep.

### Generation of iSMCs

Reprogramming of fibroblasts into the iMPCs used in this study was previously described.^[42]^ For iSMC production, 0.5 × 10^5^ or 1.0 × 10^5^ iMPCs were trypsinized and resuspended in either 10 μL F/R/C medium alone or in either 4% Matrigel (Corning, 356 237), 25 μg mL^−1^ Fibronectin (Sigma-Aldrich, F2006) or 1% Type B Gelatin (Sigma-Aldrich, G9391) diluted in low glucose DMEM (Thermo Fisher Scientific, 31 885 023). Next, resuspended iMPCs were seeded on top of the sterile PCL scaffolds and placed in a cell culture incubator (37 °C, 5% CO_2_) for 3–4 h until cells were attached to the scaffolds. The F/R/C medium was then added to each well and changed freshly every other day. All subsequent analyses (excluding the CTX injury model) were conducted 2 days after cell seeding unless otherwise indicated. For the derivation of EYFP^+^ iSMCs, iMPCs generated from mouse embryonic fibroblasts carrying a *Pax7-CreERT2; Rosa26-LSL-EYFP* were incubated in 0.1 μm 4-OHT (Sigma-Aldrich, H7904) for several days to label PAX7^+^ cells. Then, EYFP^+^/PAX7^+^ cells were FACS-purified by SH800S FACS-Sorter (Sony Biotechnology Inc), expanded in F/R/C medium and used for EYFP^+^ iSMC production.

### Generation of HSkM-Derived SMCs

HSkMs (Thermo Fisher Scientific, A12555) were cultured in growth medium and harvested upon confluency. 1.5 × 10^5^ HSkMs were resuspended in 10 μL of growth medium or 4% Matrigel and seeded onto the electrospun scaffolds. These constructs were incubated for 3–4 days in growth medium, followed by 7 days of culture in DM or F/R/C medium.

### Field Emission Scanning Electron Microscopy

For structure observation, scaffolds were cut into Ø 8 mm using a biopsy punch and fixed on metal stubs with a carbon tape coated with platinum/palladium (80/20) sputtering (Safematic, CCU-010). FE-SEM (Hitachi, SEM SU5000) was used to capture the surface topology, fiber alignment and microstructure of the scaffolds at 5 kV. For cell attachment, 5.0 × 10^4^ iMPCs were seeded onto the scaffolds and cultured for 2 days in F/R/C medium. The cells were then washed with PBS and fixed with 4% paraformaldehyde (PFA) (Alfa Aesar, 43 368) and 3% glutaraldehyde (Thermo Fisher Scientific, 119 980 250) (both suspended in PBS). As the next step, the cells were fixed with 2% osmium tetroxide in PBS (Polysciences, 23310-10) for membrane preservation, and dehydrated with a graded ethanol series ending in absolute ethanol. After dehydration, the cells were washed with hexamethyldisilazane (Sigma-Aldrich, 440 191). Finally, the samples were mounted and coated with platinum/palladium (80/20) sputtering for FE-SEM.

### Immunofluorescence Staining

At defined time points, iSMCs were fixed with 4% PFA for 10 min and washed with PBS twice. To avoid random binding of antibodies and to permeabilize the cell membrane, iSMCs were incubated for 30 min in blocking solution containing 2% BSA (AppliChem, 9048-46-8) and 0.5% Triton X-100 (Sigma-Aldrich, 9002-93-1). The blocking solution was then discarded and iSMCs were incubated for 2 h with primary antibodies diluted in blocking solution, followed by washing and addition of secondary antibodies and 4′,6-diamidino-2-phenylindole (DAPI) (1:1000, Thermo Fisher Scientific, 62 248) for 30 min. The primary antibodies used in this study include anti-Mouse Myosin Heavy Chain (1:1000, R&D Systems, MAB4470), antiHuman/Mouse/Rat/Chicken Pax7 (5 μg mL^−1^, R&D Systems, MAB1675), anti-Pax3/7 (B-5) (1:500, Santa Cruz, sc-365843), anti-Human/Mouse MyoD (5.8A) (1:200, Thermo Fisher Scientific, MA512902), anti-Myogenin (F5D) (1:250, Santa Cruz, sc-12732), and anti-𝛼-Actinin (Sarcomeric, EA53) (1:800, Sigma-Aldrich, A7811). The secondary antibodies used in this study were Goat Anti-Mouse IgG2b (Alexa Fluor 546) (1:500, Thermo Fisher Scientific, A21143), Goat anti-Mouse IgG2a (Alexa Fluor 546) (1:500, Thermo Fisher Scientific, A21133), Goat Anti-Mouse IgG1 (Alexa Fluor 647) (1:500, Thermo Fisher Scientific, A21240) and Goat Anti-Mouse IgG1 (Alexa Fluor 546) (1:500, Thermo Fisher Scientific, A21123). Fluorescence images were taken using either a confocal (Carl Zeiss, LSM 880 airyscan) or an inverted microscope (Nikon ECLIPSE Ti2).

### Quantitative Real-Time PCR

The iSMCs were placed in 1.5 mL Eppendorf tubes and directly subjected to RLT cell lysis buffer supplied in the RNeasy kit (Qiagen, 74 104) for 1 min while vortexing. As a positive control, *TA* muscles were harvested from mice, cut into small pieces and subjected to RLT buffer and vortexing for 1 min. To discard the remaining muscle fragments, the lysate was centrifugated for 3 min at the maximum speed and the supernatant was used for the remaining steps. According to the manufacturer’s instruction, the cell lysates were then used for RNA extraction and converted into cDNA by a High-Capacity cDNA Reverse Transcription Kit (Thermo Fisher Scientific, 4 368 814). The same amount of cDNA for each sample was then used for probe-based qRT-PCR. The probes used in this study were purchased from Integrated DNA Technologies (IDT, Coralville, USA) and include murine(m)*Gapdh* (Mm.PT.39a.1), m*Pax7* (Mm.PT.58.12398641), m*Notch3* (Mm.PT.58.7794053), m*Heyl* (Mm.PT.58.17090120), m*Myod1* (Mm.PT.58.8193525), m*Myog* (Mm.PT.58.30712483.gs), m*Myf6* (Mm.PT.58.33344984), and m*Myh1* (Mm.PT.58.11712984). For *Mki67*, PowerUp SYBR Green Master Mix (Thermo Fisher Scientific, A25741) was used using the following primer sequences: forward primer 5′-CAGTTTGGCGACATTAGCAGA-3′ and reverse primer 5′-GCAACTATCTTGGCAACATCCTC-3′.

### EdU Analysis

To detect proliferating cells in iSMCs, the Click-iT EdU Cell Proliferation Kit for Imaging, Alexa Fluor 647 dye (Thermo Fisher Scientific, C10340) was used according to the manufacturer’s protocol. Briefly, 10 mm EdU was added to F/R/C medium at a final concentration of 10 μm and then applied to the iSMCs for 2 h at 37 °C. Then, iSMCs were fixed with 4% PFA for 10 min and incubated in blocking solution for 20 min, followed by Click-iT reaction cocktail for 30 min at RT for EdU color development and staining. To combine an EdU analysis with conventional immunostaining, primary and secondary antibodies were applied as described in the respective “Immunofluorescence staining” method section.

### scRNA-seq Library Construction

To construct scRNA-seq libraries the cells were first detached from the PCL scaffolds by treating them with 2 mg mL^−1^ Dispase II (Thermo Fisher Scientific, 17 105 041) for 20 min at 37 °C, followed by 0.25% Trypsin-EDTA for 10 min. Scaffolds were then removed, and remaining cells were centrifugated for 5 min at 300 g. To disaggregate iMPC clumps into single cells, 0.25% Trypsin-EDTA was again applied to the cell pellet for 5 min and neutralized with F/R/C medium. Cells were then filtered through 40 μm strainer (VWR, 734-0002), resuspended in F/R/C medium and immediately used for scRNA-seq library production. The libraries were constructed using Single cell 3′ reagent kit v3.1 on 10× platform (10× Genomics, Pleasanton, CA) as detailed in a previous study.^[42]^ Libraries were sequenced in full SP flow cell of NovaSeq with paired-end 28–91 bp.

### Analysis of scRNA-seq Data

The scRNA-seq data were demultiplexed and mapped against the mouse reference genome assembly GRCm39 (GENCODE release M26) using 10× Genomics CellRanger v7.0.0.^[76]^ Downstream analysis of the resulting filtered feature-barcode count matrices was performed using the Seurat v4.2.1 pipeline.^[48,77]^ For quality control, cells with unique feature counts <250 and >9000, and mitochondrial gene counts >10% were removed. For each condition, filtered data were log normalized, scaled and the top 2000 variable features were detected. Samples were integrated based on feature anchors.^[48]^ Integrated data were scaled, and principal component analysis (PCA) was performed using the highly variable features for dimensional reduction. Cells were clustered based on the first 30 principal components (PCs) using Louvain algorithm^[78]^ with a resolution of 0.5. Clustered cells were visualized in a 2D space using the uniform manifold approximation and projection (UMAP)^[79]^ of the same PCs. For each cell cluster, conserved marker genes were identified based on the Wilcoxon rank-sum test with log2 fold-change >0.25 and adjusted p-value <0.05 cut-offs. Unsupervised single cell trajectory analysis was conducted using Monocle3 v1.2.9^[80,81]^ and cells were ordered in pseudotime along the learned trajectory at the conditional level with cluster 1 functioning as the root.

### Cardiotoxin Injury

To induce muscle injury in iSMCs, 5.0 × 10^4^ iMPCs were seeded onto PCL scaffolds and incubated in F/R/C medium containing 2 μm CTX (Latoxan, L8102) 4 h after cell attachment. On the next day, F/R/C medium containing CTX was discarded, and fresh medium was applied. For the second injury, 4 days after the first CTX removal, iSMCs were treated with 4 μm CTX in F/R/C medium for 24 h and then incubated in fresh medium for recovery.

### Assessment of PAX7^+^ Cell Viability after Cardiotoxin Injury

5.0 × 10^4^ iM-PCs carrying *Pax7-CreERT2; Rosa26-LSL-ntdTomato* cassettes were seeded onto PCL scaffolds. A 2 μm CTX and 0.1 μm 4-OHT were added to the iSMCs after 4 h of cell attachment. On the next day, CTX-treated iSMCs were washed once with PBS and incubated in F/R/C medium containing Calcein-AM supplied from a Live/Dead assay kit (Thermo Fisher Scientific, L3224) for 10 min prior to analysis.

### Statistical Analysis

Statistical analysis was conducted using the software GraphPad Prism as indicated in each respective figure legend.

## Supplementary Material

Supplementary Materials

## Data Availability

The data that support the findings of this study are available from the corresponding author upon reasonable request. The scRNA-Seq datasets reported in this study are available at Gene Expression Omnibus (GEO) repository (GSE222162).
