# Supplementary material for: A Self-Renewing Biomimetic Skeletal Muscle Construct Engineered using Induced Myogenic Progenitor Cells
Source: Adv Funct Mater. Author manuscript; Available in PMC 2025 Jul 17. (PMC7617902; doi:10.1002/adfm.202300571)
Supplement: Supplementary Materials [file EMS205878-supplement-Supplementary_Materials.pdf]

ADVANCED
FUNCTIONAL
MATERIALS

Supporting Information

for *Adv. Funct. Mater.*, DOI 10.1002/adfm.202300571

A Self-Renewing Biomimetic Skeletal Muscle Construct Engineered using Induced Myogenic Progenitor Cells

Inseon Kim, Seunghun S. Lee, Adhideb Ghosh, Stephen J. Ferguson and Ori Bar-Nur**

Supporting Information

A Self-Renewing Biomimetic Skeletal Muscle Construct Engineered using Induced Myogenic Progenitor Cells

Inseon Kim, Seunghun S. Lee, Adhideb Ghosh, Stephen J. Ferguson and Ori Bar-Nur**

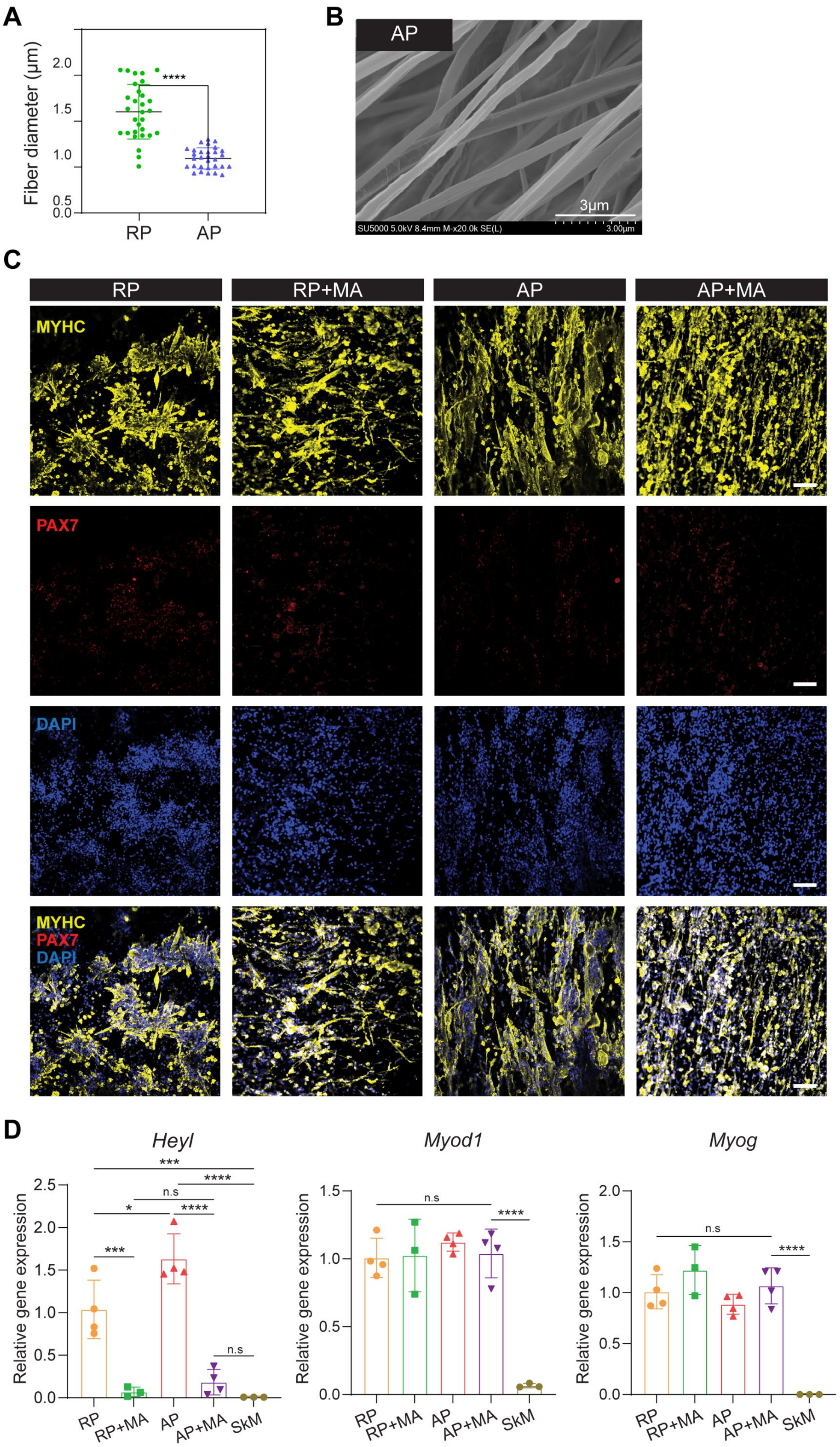

A
Fiber diameter (μm)
2.0
1.5
1.0
0.5
0.0
****
RP
AP
B
AP
3μm
SU5000 5.0kV 8.4mm M-x20.0k SE(L)
3.00μm
C
RP
RP+MA
AP
AP+MA
MYHC
PAX7
DAPI
MYHC
PAX7
DAPI
D
Heyl
Myod1
Myog
Relative gene expression
***
****
n.s
*
****
***
n.s
RP
RP+MA
AP
AP+MA
SkM
n.s
****
n.s
****

Figure S1. (A) Diameter measurement of electrospun PCL nanofibers. The data are shown as means ± S.D (n=30). Statistical significance was determined by a two-tailed unpaired t-test (****p<0.0001). **(B)** A FE-SEM image of AP scaffolds at a high magnification. **(C)** Representative immunofluorescence images of iSMCs stained for MYHC (yellow), PAX7 (red), and DAPI (blue) for the indicated conditions. Please note that images with the same field of view at higher magnification are provided in Figure 2D. Scale bar, 100 µm. **(D)** Graphs showing qRT-PCR analysis of the indicated genes. The data are shown as means ± S.D (n=3-4 independent experiments). Statistical significance was determined by one-way ANOVA (*p<0.05, n.s = nonsignificant).

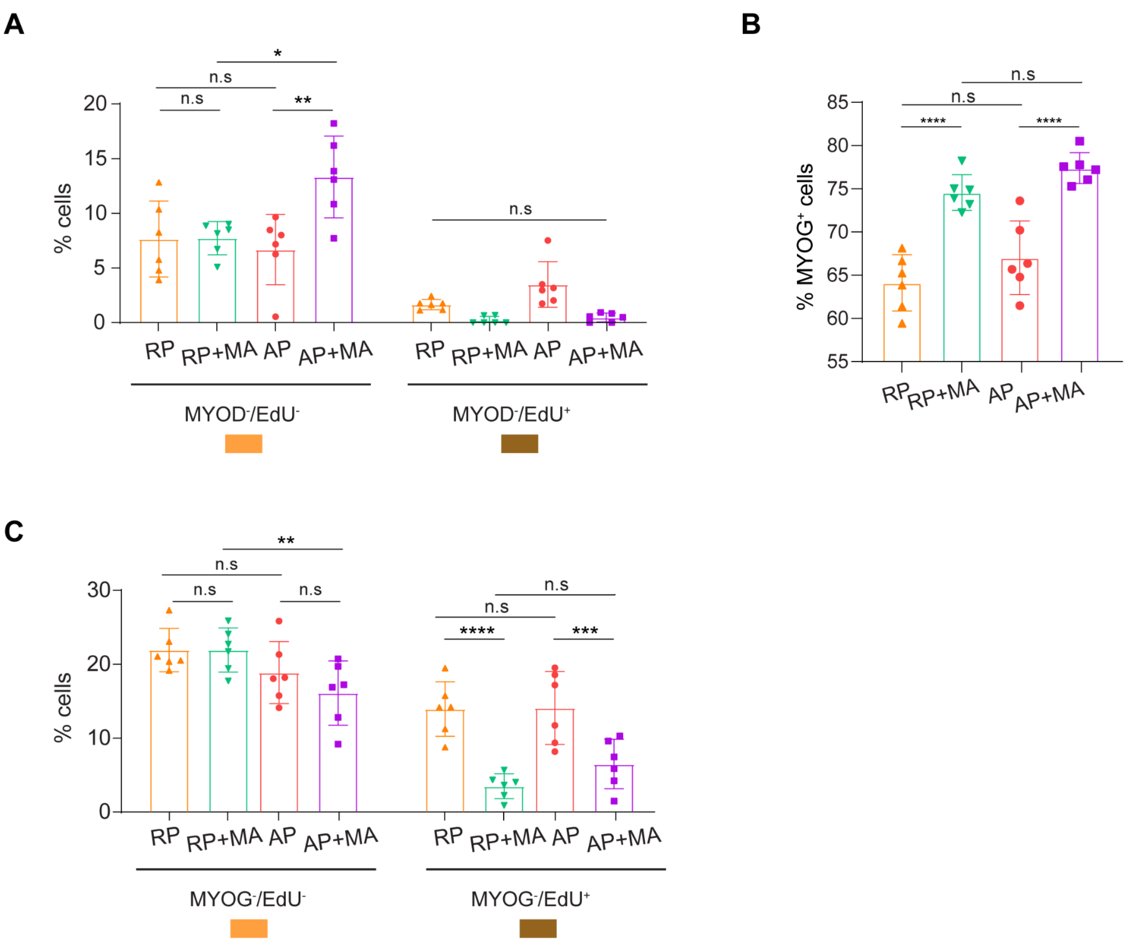

Figure S2. (A) Graphs showing quantification of the images presented in Figure 3B. The data are shown as means ± S.D (n=6, 3 different images from 2 independent experiments were quantified). Statistical significance was determined by two-way ANOVA (*p<0.05, **p<0.01, n.s = nonsignificant). **(B)** A graph showing quantification of the MYOG positive cells shown in Figure 3F. The data are shown as means ± S.D (n=6, 3 different images from 2 independent experiments were quantified). Statistical significance was determined by two-way ANOVA (****p<0.0001, n.s = nonsignificant). **(C)** Quantification of the images shown in Figure 3F. The data are shown as means ± S.D (n=6, 3 different images from 2 independent experiments were quantified). Statistical significance was determined by two-way ANOVA (**p<0.01, ***p<0.001, ****p<0.0001, n.s = nonsignificant).

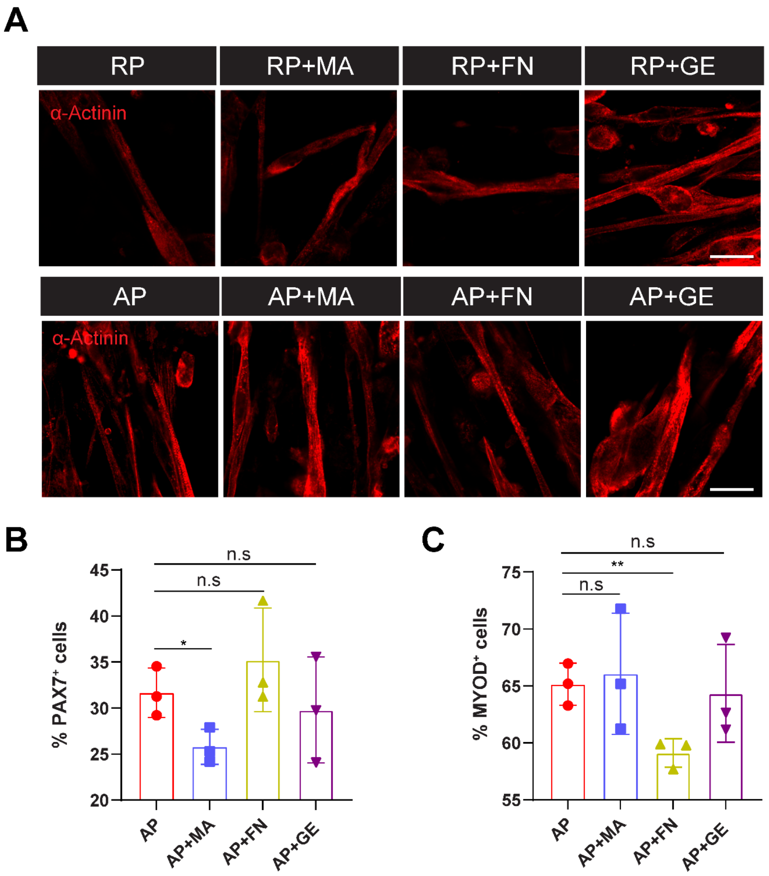

Figure S3. (A) Zoom-in images of α-ACTININ^{+} myotubes generated using the indicated conditions. Note that AP+MA, RP and RP+MA conditions exhibit weak striation. Scale bar, 25 μm. **(B)** A graph showing quantification of the PAX7^{+} cells shown in Figure 4E. The data are shown as means ± S.D (n=3). Statistical significance was determined by a two-tailed unpaired t-test (*$p<0.05$, n.s = nonsignificant). **(C)** A graph showing quantification of MYOD^{+} cells shown in Figure 5E. The data are shown as means ± S.D (n=3). Statistical significance was determined by a two-tailed unpaired t-test (**$p<0.01$, n.s = nonsignificant).

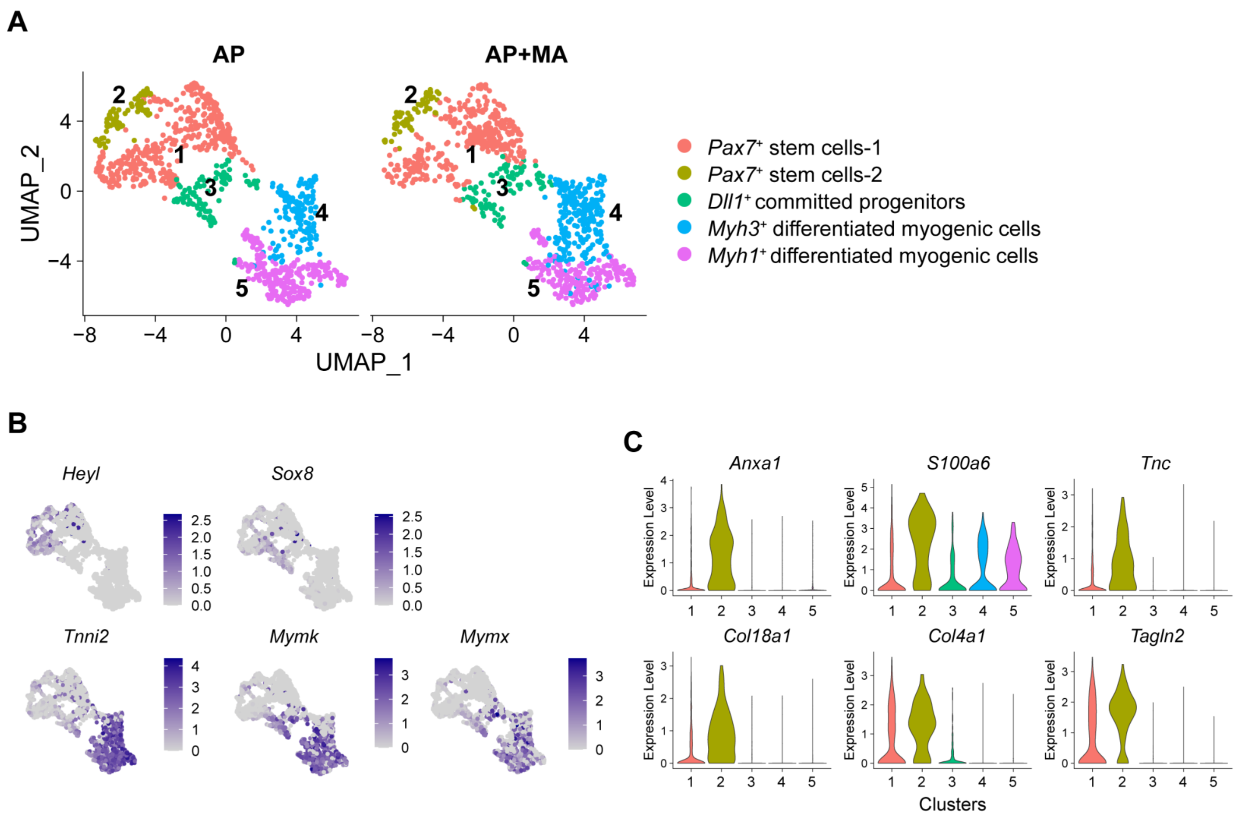

Figure S4. (A) UMAP projections based on scRNA-seq data of the conditions AP and AP+MA. Cells are colored by clusters. **(B)** Feature plots showing the expression level of the indicated genes. **(C)** Violin plots showing the expression level of extracellular matrix-related genes that are enriched in cluster 2.

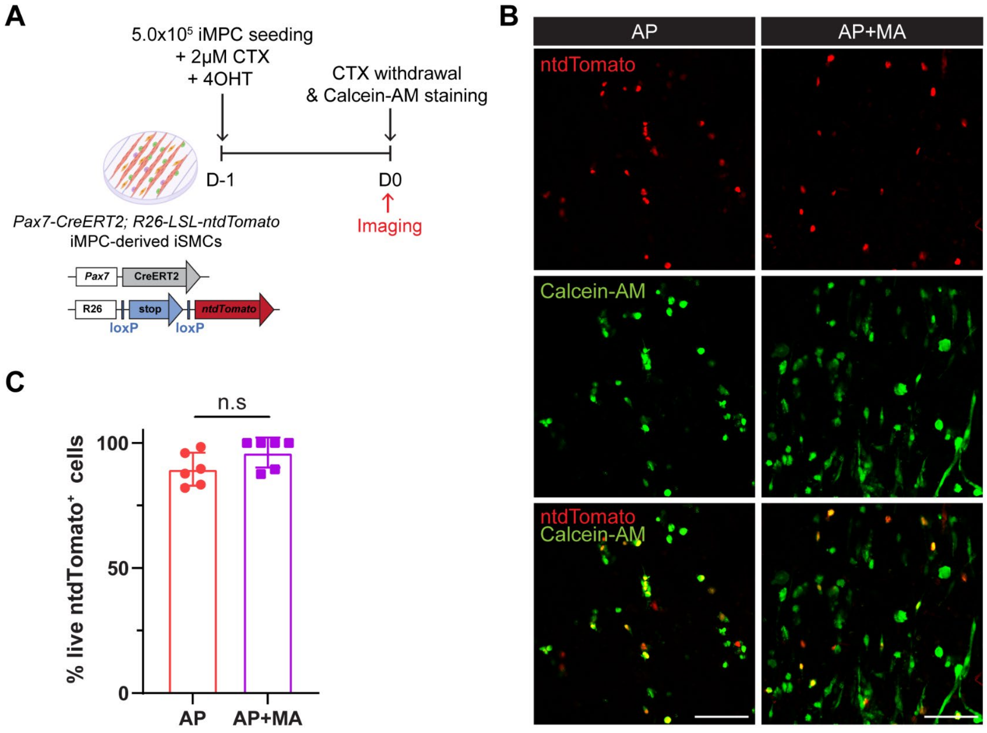

Figure S5. **(A)** Schematic of experimental design. **(B)** Representative fluorescence images of iSMCs 1 day after CTX administration. The ntdTomato^{+} and Calcein-AM^{+} cells represent PAX7^{+} and live cells, respectively. Scale bar, 100 μm. **(C)** A graph showing quantification of (B).
